# A comparison of the nutritional content of processed foods available on the French market, according to the type of brand, and potential impact on nutrient intakes—An Oqali study

**DOI:** 10.1002/fsn3.655

**Published:** 2018-06-14

**Authors:** Cécile Perrin, Charlène Battisti, Amélie Chambefort, Olivier Digaud, Barbara Duplessis, Jean‐Luc Volatier, Julie Gauvreau‐Béziat, Céline Ménard

**Affiliations:** ^1^ French Agency for Food Environmental and Occupational Health Safety Risk Assessment Department Maisons‐Alfort France

**Keywords:** nutrient content, nutrient intake, nutritional labeling, processed foods, types of brands

## Abstract

The French Observatory of Food Quality (Oqali) aims to collect all nutrition data provided on processed food labels, at the level of brand products, in order to monitor reformulation and nutrition labeling changes over time. This work aimed to make a cross‐sectional comparison of the nutrition content of processed foods on the French market, according to their type of brand (national brands, retailer brands, entry‐level retailer brands, hard discount, and specialized retailer brands), and to study the potential impact of the differences observed on simulated nutrient intakes. A total of 16,453 branded processed foodstuffs were considered, collected between 2008 and 2011 and divided into 24 food sectors. Labeled nutrition values were compared between types of brands by family of products. Nutrition values were matched with consumption data from the French Individual and National Study on Food Consumption (INCA 2) (Afssa, 2006–2007) to determine whether the nutrition differences underlined were magnified or diminished when crossing them with consumption data. Only isolated differences in nutrient contents between types of brands could be highlighted. In the case of a theoretical and exclusive consumption of processed foodstuffs from one specific type of brand, protein intakes from first‐price products (entry‐level retailer brands and hard discount) appeared to be significantly lower than the ones from national or retailer brand products. The absence of systematic differences in the nutrition contents of processed foods from various types of brands is an encouraging result when considering social inequalities and nutrition. As protein intakes in France are currently above recommended levels (Afssa, 2007), consumption of first‐price foodstuffs does not imply any risk of deficiency for French consumers.

## 1 INTRODUCTION

Due to rising differences in overweight and obesity prevalence between social groups (Afssa, 2007; Darmon & Drewnowski, 2008; Lee, Ralston, & Truby, 2011; Maillot, Darmon, Vieux, & Drewnowski, 2007; Roos et al., 2001), especially among children (Anses, 2012; Beydoun, Powell, Chen, & Wang, 2011; Pilgrim et al., 2012), nutritionists and stakeholders have been increasingly in need of accurate data on the link between the nutrition quality of processed foods (Weaver et al., 2014) and their price/type of brand (for instance retailer or national brands).

Several recent articles and reports have endeavored to compare the nutrition quality of products from different types of brands and various food sectors. They focussed both on the labeling of nutrition information on packages and on the nutritional composition of processed foods according to the type of brand. Several studies have shown that entry‐level foodstuffs (as opposed to core‐market and high‐end foodstuffs, belonging to both retailer brands or national brands) displayed nutrition information on their packages with a lower frequency than core‐market and high‐end foodstuffs (CLCV, 2009; CNA, 2010; Guibert, 2007; Joly et al., 2007). Furthermore, the review published in 2010 by the French National Food Council (CNA) concluded that, considering the nutrition values labeled, no significant difference in the nutrition quality could be highlighted between entry‐level products (corresponding to hard discount and entry‐level retailer brand products) on the one hand and retailer or national brand ones on the other hand. These observations were based both on Oqali reports published until then (Breakfast cereals 2008 (OQALI, 2008a), Cakes and biscuits 2008 (OQALI, 2008b) and Fresh dairy products, and similar 2008–2009 (OQALI, 2009) and on a bibliographic search. The results of the few Oqali reports processed on this occasion (Oqali website) showed only isolated differences in the nutrition content between types of brands. The French consumers’ association CLCV (Consumption, Housing, and Living Environment) described similar results in 2009 (CLCV, 2009). In their study entitled “First prices and quality” published in 2010, the Belgian CRIOC (Information and Research Centre for Consumer associations) could not identify any causal link between price and nutrition quality either (CRIOC, 2010). However, these findings were generally based on a limited range of products and categories (Cooper & Nelson, 2003; Faulkner, Livingstone, McCaffrey, & Kerr, 2014; Waterlander, Van Kouwen, & Steenhuis, 2014). The results presented in this article thus aim to strengthen these observations by covering almost all of the processed foods available on the French market.

The French Observatory of Food Quality (Oqali) (Oqali website) is a project set‐up in 2008 by the Ministries in charge of Agriculture, Health, and Consumer Affairs. It is implemented both by the French Agency for Food, Environmental, and Occupational Health and Safety (Anses) and the French National Institute for Agricultural Research (INRA) (Menard et al., 2011). The Observatory collects and analyses almost all nutrition data provided on labels of processed foodstuffs, at the level of branded products. These analyses enable the monitoring of changes in nutrition labeling (nutrition information and composition) of processed foods over time (Goglia et al., 2010; Menard et al., 2012). Consequently, all labeling parameters provided on packaging (nutrition labeling, nutrition and health claims, serving sizes, etc.) are collected. Socioeconomic parameters such as market shares and types of brands are also taken into account (Menard et al., 2011). With more than 40,000 food items in its database, almost all types of processed foods are now monitored by Oqali. This work aimed to give a thorough, cross‐sectional comparison of the nutrition content of processed foods on the French market, according to their type of brand, and to study the potential impact of the differences observed on simulated nutrient intakes.

## 2 MATERIAL AND METHODS

### 2.1 Data sources

A total of 16,453 branded processed foodstuffs, divided into 24 food sectors (Table 1) and 355 product‐families, were considered in this study. The products were collected between 2008 and 2011, depending on the food sector. Sales volume data were provided by Kantar Worldpanel^1^ in accordance with the years of collection of Oqali samples to estimate the market coverage of the processed foods collected by Oqali.

**Table 1:** Food sectors considered in the study, with their associated number of products, year of data collection and estimated market coverage per food sector, and type of brand

				Estimated market coverage for^a^					
Food sector	Number of families by food sector	Number of foodstuffs taken into account	Year of data collection	Total food sector (%)	National brands (%)	Retailer brands (including entry‐level retailer brands) (%)	Hard discount (%)	Specialized retailer brands (%)	Studied nutrients^b^
Bread products	41	620	2009	57	45	66	84		E; F; C; P; SFA; S; DF; Na
Breakfast cereals	11	336	2008	75	80	57	85		E; F; C; P; SFA; S; DF; Na
Cakes and biscuits	85	1,756	2008	70	86	65	79		E; F; C; P; SFA; S; DF; Na
Canned fruits	4	184	2009	69	60	52	45		E; C; S; DF
Cereal bars	5	174	2010–2011	79	89	85	80		E; F; C; P; SFA; S; DF; Na
Chocolate products	23	630	2009	68	73	62	83		E; F; C; SFA; S; DF
Cold sauces	10	500	2011	76	74	77	84		E; F; C; SFA; S; Na
Crackers	13	600	2009	49	42	52	65		E; F; C; P; SFA; S; DF; Na
Delicatessen meat	41	1,166	2010	66	59	74	81		E; F; P; SFA; Na
Dessert mixes	14	135	2009	67	63	85	74		E; F; C; P; SFA; S; DF; Na
Fresh dairy products and similar	18	1,599	2008–2009	66	70	64	58		E; F; C; SFA; S
Fresh Delicatessen products	57	2,009	2008‐2009‐2010‐2011	66	47	87	90		E; F; C; P; SFA; S; Na
Frozen pizzas	8	213	2010	62	67	67	43	50	E; F; C; P; SFA; S; DF; Na
Fruit juices and nectars	5	889	2009–2010	55	38	68	53		E; C; S
Fruit purees, compotes, and desserts	6	440	2009	68	80	65	62		E; C; S; DF
Hot sauces	22	294	2010	77	85	79	71		E; F; C; P; SFA; S; DF; Na
Ice creams and sorbets	18	1,476	2010–2011	67	72	60	41	80	E; F; C; SFA; S
Jams	5	339	2009	65	64	53	47		E; C; S; DF
Margarines	3	95	2011	82	85	72	84		E; F; SFA; Na
Processed potato products	14	629	2011	76	65	81	83	87	E; F; C; SFA; S; DF; Na
Ready‐to‐eat canned meals	25	765	2010	71	67	84	80		E; F; C; P; SFA; S; DF; Na
Soft drinks	18	760	2009–2010	78	83	75	59		E; C; S
Soups and broths	16	540	2011	77	78	84	86		E; F; C; P; S; DF; Na
Syrups	3	304	2009–2010	69	65	76	57		E; C; S; DF
Total	465	16,453	2008–2011	69	68	70	70	72	

a Sales volume ratio of products collected by Oqali to total sales identified by Kantar Worldpanel.

b E, energy value; F, fat; C, carbohydrates; P, proteins; SFA, saturated fatty acids; S, sugars; DF, fibers; and Na, sodium.

All nutrition data labeled on the food packages were entered and codified in the Oqali database: general information describing the product (name, type of brand, barcode, net weight, etc.), nutrition labeling (claims, serving sizes, guideline daily amounts, etc.), the list of ingredients, and the nutrition values labeled as well. Only one package size for each food product was included in the analysis. This was to ensure that frequencies were not biased by products with multiple pack sizes. Inside each food sector, food products were divided into product‐families (e.g., among Cakes and biscuits food sector, product‐families of chocolate biscuits, or fruit biscuits were distinguished) and belonged to one of the following types of brands: national brands, retailer brands, entry‐level retailer brands, hard discount, or specialized retailer brands. National brands correspond to products distributed nationally under a brand name owned by a food manufacturer (national or international). Retailer brands cover products carrying the brand of the retailer rather than the producer and sold only in their own supermarket chain. Entry‐level retailer brand products correspond to first‐price retailer brand products: their plain packaging often reveals this positioning. Hard discount store brands are products sold at prices below the typical market value, with a focus on price rather than service. Specialized retailer brands correspond to frozen products sold in freezer centers and by home delivery suppliers.

### 2.2 Nutrition values studied

Eight components were considered in the study: energy value, fats, carbohydrates, proteins, saturated fatty acids, sugars, fibers, and sodium. For each of them, the associated nutrition values considered were those labeled on foodstuffs. For the 24 food sectors considered, only relevant nutrients were studied and tested (for instance, Delicatessen meat was only tested for energy value, proteins, fats, saturated fatty acids, and sodium, Table 1).

For each nutrient, a comparison was made at product‐family level between types of brands. Mean contents (with standard deviation) were calculated for each triplet: nutrient/product‐family/type of brand. Insufficient numbers of products (i.e., less than or equal to 2) for a given triplet were not considered (an exception was made for the calculation of the maximal differences between type of brand mean content). First, a nonparametrical Kruskal–Wallis test enabled us to identify, within each product‐family studied and for a given nutrient, whether there was at least one type of brand which had nutrition values that were different from those of the other types of brands. Then, only for significant results, paired statistical tests (Wilcoxon with Bonferroni correction) were undertaken to identify which type of brand differed from which other one.

### 2.3 Simulation of potential impact on nutrient intakes

The nutrition values studied were also matched with consumption data from the French Individual and National Study on Food Consumption (INCA 2) (Afssa, 2006) in order to evaluate whether the nutrition differences reveal within product‐families between types of brands were magnified or diminished by crossing them with consumption data.

Three types of brands were distinguished: national brands, retailer brands, and first‐prices (gathering hard discount and entry‐level retailer brands). These two last types of brands were gathered to obtain higher numbers of products, as entry‐level retailer brands had too few products to be studied individually.

The INCA 2 study took place between 2006 and 2007 and included the intakes of 1,918 adults (from 18 to 79 years old), 570 children (from 3 to 10 years old), and 874 teenagers (from 11 to 17 years old). Participants were selected according to a three‐stage random sampling, stratified according to the degree of urbanisation and the location. Food intakes were collected by means of a seven‐day intakes diary recording the nature of the foodstuffs eaten and the corresponding quantities eaten at a specific time. For each record, the place of consumption and the type of food eaten (processed or homemade) were also recorded.

#### 2.3.1 Matching food products from the Oqali database with INCA 2 nomenclature foodstuffs

The INCA 2 food nomenclature describes 1,342 generic foodstuffs (i.e., not at brand level) divided into 43 food groups. Each Oqali branded product had thus to be associated with one of these 1,342 INCA 2 foodstuffs in order to link Oqali nutrition values to INCA 2 consumption data. Following this matching, only INCA 2 foodstuffs associated with at least one Oqali product of each type of brand (national, retailer, and first‐price brand) could be taken into account, so that the corresponding nutrient intakes could be compared afterward.

#### 2.3.2 Calculation of weighted nutrition values per type of brand

For each INCA 2 foodstuff/type of brand pair, average labeled nutrition values weighted by the volumes of sales were calculated. Sales volume data were provided by Kantar Worldpanel in accordance with the years of collection of Oqali samples, although not all Oqali products could be associated with a sales volume (for instance due to a lack of information linking Oqali products to Kantar Worldpanel references). If an Oqali product had no sales volume associated and was the only product from its type of brand that matched the INCA 2 foodstuff, then it was assumed that its market share was equal to 100% for this INCA 2 foodstuff/type of brand pair. This case apart, only Oqali products with sales volume were considered to calculate average food composition.

For the food sectors studied, it was assumed that Oqali had collected data covering all food products supplied on the French market. Consequently, it was considered that the sum of sales volumes of Oqali products matched with a given INCA 2 foodstuff corresponded to the total sales volume of the INCA 2 generic foodstuff in question. For each Oqali product A from the type of brand B matched with the generic INCA 2 foodstuff C, the associated market share was calculated according to Formula (1):

(1) $$\begin{array}{cc} & \text{Market share (A)}=\text{Sales volume (A)}/\sum (\text{Sales volumes of}\\ & \text{Oqali products from type of brand B matched}\\ & \text{with INCA 2 foodstuff (C))} \end{array}$$

For each INCA 2 foodstuff considered, weighted average nutrition values per type of brand could then be calculated.

Moreover, weighted average nutrition values were also calculated for each INCA 2 foodstuff considered, taking into account all Oqali products matched with this INCA 2 foodstuff, regardless of the type of brand. This time, the market shares weighting these average values were calculated on the basis of the whole set of Oqali products associated with a given INCA 2 foodstuff, for all types of brands gathered.

#### 2.3.3 Study of the impact of brand loyalty on nutrient intakes

Three types of scenario were studied: (a) three maximalist scenarios of consumers that are exclusively loyal to a specific type of brand; (b) three scenarios of consumers that are highly loyal to a specific type of brand; (c) one scenario of consumers that buy all types of brands.

The average nutrition values used differed depending on the scenario:

- Maximalist scenarios: these cover use of the weighted average nutrition values per type of brand, which means a theoretical consumer only eats products from this type of brand (national brands, retailer brands, or first‐price brands)
- High‐loyalty scenarios: this time, the loyalty of the consumers is not exclusive. This theoretical consumer picks products from his/her favorite type of brand in 60% of cases, products from one of the other types of brands in 20% of cases and from the third one in the last 20% of cases. Combined weighted average nutrition values were calculated by means of Formula (2) with the example of a high loyalty to national brands:

(2) $$\begin{array}{cc} & \text{Average nutrition values for a high loyalty to national brands}\;\\ & =\;60\%\;\text{national brands weighted average nutrition values}\;\\ & \;\;\;\;\;+\;20\%\;\text{retailer brands weighted average nutrition values}\;\;\\ & \;\;\;\;\;+\;20\%\;\text{first price‐weighted average nutrition values} \end{array}$$

- Scenario of average intakes from all types of brands: in this case, the weighted average nutrition values used, took into account all the products matched with a given INCA 2 foodstuff, regardless of the type of brand. They reflected the whole market for this INCA 2 foodstuff.

#### 2.3.4 Nutrition intake calculation

Weighted average nutrition values described previously (depending on the scenario considered) were then combined with consumption data from the INCA 2 study. A maximalist approach was chosen: The circumstances of consumption records were not taken into account (i.e., place of consumption, homemade/processed foods), and the nutrition values calculated were combined with all consumption records linked to a given INCA 2 foodstuff in order to exacerbate the impact on related nutrition intakes. This may nonetheless have introduced a bias considering the existence of potential differences in consumption profiles according to the different circumstances of a meal.

Final simulated intakes according to the various scenarios were then calculated by age and sex population. The design of the INCA 2 study was taken into account in this calculation.

#### 2.3.5 Statistical tests undertaken

For each nutrient studied, statistical tests were undertaken to determine whether there were some significant differences in nutrition intakes among a given population, according to the different scenarios. An analysis of variance (ANOVA) was performed, with the intake of each nutrient as the variable to be explained and the type of brand as the explanatory variable. If the ANOVA revealed significant differences between nutrition intakes (significance level of 5%), then Tukey post hoc comparisons were undertaken to identify which types of brands differed from one another.

## 3 RESULTS

### 3.1 Oqali food sectors and market coverage

Table 1 gives the distribution of the 16,453 branded foodstuffs, collected according to their food sector. The spread in the years of collection (from 2008 to 2011) may introduce a bias as to the indicators followed by Oqali, considering the changes in food regulations over the past few years (Food Information for Consumer Regulation (EU) n° 1169/2011 (European Parliament and Council EU, 2011), discussions around the set‐up of new regulations for health claims, etc.).

Table 1 also shows the estimated market coverage at several levels: for the entire food sector and for each type of brand. The estimated market coverage for Oqali samples at food sector level varied from 49% (Crackers‐2009) to 82% (Margarines‐2011). The estimated market coverage per type of brand was also calculated, and they were all around 70%: 72% for specialized retailer brands, 70% for both hard discount and retailer brands (including entry‐level retailer brands), and 68% for national brands. However, Table 1 shows that there might sometimes be a bias in the results observed, due to uneven market coverage between types of brands for a given food sector.

### 3.2 Comparison of the nutrition values labeled between types of brands

It should first be noted that much more nutrition data were available for national and retailer brand products (compare to hard discount and entry‐level retailer brands), mainly thanks to their higher number of products on the market. Moreover, national and retailer brand products had the highest frequencies of nutrition labeling^2^ (94% for retailer brands, 90% for national brands versus 87% for hard discount, and 71% for entry‐level retailer brands) and also of detailed nutrition labeling^3^ (76% for retailer brands, 61% for national brands versus 41% for hard discount, and 28% for entry‐level retailer brands) (Perrin et al., 2017). This is why comparisons were mainly feasible between retailer brands and national brands. The fact that more significant differences might be found between these two types of brands must then be qualified by the lower amounts of data available for other types of brands, causing statistical tests to be less powerful as far as the latter are concerned.

Table 2 summarizes, for the eight nutrients studied, the amount of data available for the comparative study of nutrition values, the number of relevant product‐families (i.e., relevant for the nutrient of interest) that were actually tested and the percentage within these relevant product‐families where significant differences were found in the nutrition content between types of brands. The low percentages noted (between 2% and 8%) show that only isolated and nonsystematic differences could be determined, when considering the nutrition values labeled on processed foods on the French market. No cross‐sectional tendency was found among the 24 sectors studied in this comparative study between types of brands.

**Table 2:** Number of products labeling the different components studied, number of relevant families associated, and percentage of families where significant differences between types of brands were noted

Component	Number of products with a nutrient content labeled	Number of product‐families relevant for study of the nutrient	Percentage (number) of relevant product‐families with significant differences in nutritional content between types of brands
Energy value	14,378	353	8% (*n* = 28)
Fats	14,382	317	7% (*n* = 21)
Carbohydrates	14,386	20	8% (*n* = 24)
Proteins	14,382	197	8% (*n* = 15)
Saturated fatty acids	10,162	270	6% (*n* = 15)
Sugars	10,179	320	5% (*n* = 15)
Fibers	10,160	235	2% (*n* = 5)
Sodium	10,254	287	3% (*n* = 9)

Significant differences in fat content between types of brands were found for only 7% of the 317 relevant product‐families studied (Table 2). These were isolated observations, and the amplitude of the differences was low: The maximal amplitude noted between types of brands was for the Pork lardons family within the Delicatessen meat sector with a 9.9 g/100 g gap between the average fat contents of entry‐level retailer brand (29.8 g/100 g) and national brand products (19.9 g/100 g) (data not shown). A parallel can be drawn with the difference in average energy values, also observed for these two types of brands for the Pork lardons family (325 kcal/100 g for entry‐level retailer brand products and 249 kcal/100 g for national brand ones) and in average protein contents (14.0 g/100 g for entry‐level retailer brands and 17.0 g/100 g for national brands) (data not shown).

Significant differences between types of brands were found for only 5% of the 320 relevant product‐families considered for the study of sugar content. The maximal difference observed between average sugar content by type of brand was related to the Ice cream coupes family (within the Ice creams and sorbets sector) with a 8.8 g/100 g gap between national brand products (17.6 g/100 g) and entry‐level retailer brand ones (8.8 g/100 g) (data not shown). However, it should be noted that only one datum was available for entry‐level retailer brands.

Differences between types of brands were found for only 3% of the 287 relevant product‐families tested for differences in sodium content. The widest gaps observed between average sodium content by type of brand were 0.90 g/100 g for the Peanuts family (within the Crackers sector) between hard discount products (1.80 g/100 g) and national brand ones (0.90 g/100 g), and 0.86 g/100 g for Aperitif crackers family (within the Crackers sector) between hard‐discount products (1.26 g/100 g) and entry‐level retailer brand ones (0.40 g/100 g) (data not shown).

Differences between types of brands were found for only 8% of the 197 relevant product‐families tested for differences in protein content. Concerning Delicatessen meat and for three product‐families among the 32 for this food sector (Raw ham, Pork lardons, and Superior cooked ham), hard discount products had average protein contents lower than the ones of national brands and retailer brands (data not shown). A significant difference of 5.2 g/100 g was noted for average protein content (data not shown) for Raw ham between retailer brand (28.8 g/100) and hard discount (23.6 g/100 g).

Product‐families from the Ice creams and sorbets sector showed significant differences in the nutrition content between types of brands for several components: energy value, fats, carbohydrates, saturated fatty acids, and sugars. Indeed, within a single product‐family, national brand products and specialized retailer brand ones were associated with higher average contents than other types of brands, due to more elaborate and thus richer recipes, with frequent inclusion of sauces, biscuits, and nuts.

These various results all point in the same direction and show that dietary nutrient quality is not negatively affected by an increased consumption of first‐price foodstuffs.

### 3.3 Simulation of the potential impact of nutrition differences between types of brands on nutrient intakes

#### 3.3.1 INCA 2 foodstuffs considered and nutrition data available

Following the matching of INCA 2 foodstuffs and Oqali products, 343 INCA 2 foodstuffs—of 1,342 in the whole INCA 2 nomenclature—had been associated with at least one Oqali product from each type of brand studied (national brands—retailer brands—first‐price brands) and could thus be taken into account. As shown in Table 3, they belonged to 26 food groups from the INCA 2 nomenclature and mainly to Chocolate (14 INCA 2 foodstuffs taken into account out of 16 for the whole nomenclature, i.e., 88%), Biscuits (85%), Ice cream and iced desserts (82%), and Mashed and cooked fruits (82%). It should be remembered that Oqali aims to monitor processed foodstuffs only, which explains the low percentages of INCA 2 foodstuffs studied for some food groups, as these groups also include mainly nonprocessed or homemade foods.

**Table 3:** Number of INCA 2 foodstuffs associated with Oqali products, by food group

INCA 2 food groups	Number of INCA 2 foodstuffs studied inside the group	Percentage of foodstuffs studied (%)	INCA 2 food groups	Number of INCA 2 foodstuffs studied inside the group	Percentage of foodstuffs studied (%)
Chocolate	14	88	Meat products	21	38
Biscuits (savory and sweet), bars	29	85	Pastries and cakes	17	37
Mashed and cooked fruits	9	82	Croissant‐like pastries	4	33
Ice cream and iced desserts	9	82	Sandwiches, hamburgers	9	31
Cream desserts	25	78	Dried fruits, nuts, and seeds	7	28
Pizzas, salty pastries	15	71	Condiments and sauces	17	27
Nonalcoholic beverages	38	63	Sugar and confectionery	6	26
Breakfast cereals	15	63	Pasta	1	20
Potatoes	7	58	Margarines	5	19
Soups	11	58	Other hot drinks	1	9
Dairy products	29	48	Poultry and game	2	9
Bread and bread products	11	44	Vegetables (excluding potatoes)	8	8
Mixed dishes	32	41	Foods for specific needs	1	6

For these 343 INCA 2 foodstuffs, the comparative study of nutrition intakes concerned energy value, fats, carbohydrates, and proteins. Indeed, as considered products were collected between 2008 and 2011, when EU regulation n°1169/2011, which makes detailed nutritional labeling mandatory, was not yet in force, too few products labeled Big 8 components (energy, proteins, carbohydrate, sugars, fat, saturated fat, fibers, and sodium) on their back‐of‐pack nutrition tables. Moreover, there are great differences in detailed nutrition labeling between types of brands (Perrin et al., 2017) as evoked before (OQALI, 2015).

#### 3.3.2 Part of the total diet studied

Table 4 shows the corresponding daily amounts of foods consumed for the 343 foodstuffs and associated consumption records taken into account in this study. It enables us to put into perspective the intakes considered (due to these 343 foodstuffs) that account for between 19% and 43% of the total diet (with and without water) depending on the population considered. This can be explained both by the fact that raw food groups are not studied by Oqali (Table 3) and also by the methodology requiring the study of only INCA 2 foodstuffs matched with at least one product from each type of brand. In terms of energy intake, intakes covered by the study corresponded to 35% of men's total energy intakes without alcohol, taken from the INCA 2 study (Afssa, 2006) (with reference for men's average energy intake without alcohol at 2,348 kcal/day).

**Table 4:** Daily food quantities consumed for different populations for the 343 INCA 2 foodstuffs studied and percentage of these consumed quantities in the total food diet (with or without water), according to the INCA 2 study

			Food quantities consumed (g/day), related to the 343 foodstuffs studied, for all consumption records associated				% of the food quantities consumed in total diet according to the INCA 2 study (%)	% of the food quantities consumed in total diet WITHOUT WATER according to the INCA 2 study (%)
Population	Gender	*N*	Mean	Min	Max	*SD*		
Adults (*n* = 1,918)	Male	776	555.2	1.4	2,434.5	11.3	19	26
	Female	1,142	478.7	42.5	3,223.3	8.1	19	27
Teenagers (*n* = 874)	Male	408	646.9	80.1	1,752.0	15.2	31	44
	Female	466	532.3	29.4	1,793.1	10.4	30	43
Children (*n* = 570)	Male	276	523.3	91.4	1,997.2	9.8	31	42
	Female	294	487.1	136.5	1,457.4	10.7	31	42

#### 3.3.3 Scenarios of simulated nutrient intake

Table 5 shows the results obtained for adults by comparing energy value, fats, carbohydrates, and protein intake in the case of the maximalist scenarios (scenario [a]) of total loyalty to a specific type of brand by a hypothetical consumer. The last column of Table 5 shows the results obtained in the case of average market intake for all types of brands considered (scenario [c]): These figures serve as a reference. Intakes relating to the 343 INCA 2 foodstuffs considered are compared between an individual eating only average national brand products, another one eating only average retailer brand products, a third one eating only average first‐price products, and a last one eating average products with a composition representative of the total market supply (composed of retailer brand, national brand, and first‐price products). For significant tests (*p*‐value <.05), mean values with no common letter (“a” on one hand and “b” on the other for instance) are statistically and significantly different, whereas the ones with a common letter are not significantly different.

**Table 5:** Comparison of adult daily intakes related to the consumption of 343 INCA2 foodstuffs for four components, between the three maximalist scenarios of total loyalty to a specific type of brand and for average intake considering all types of brands

Adults (*n* = 1,914)			Intakes for national brand products, total loyalty Scenario A1				Intakes for retailer brand products, total loyalty Scenario A2				Intakes for first‐price products, total loyalty Scenario A3				Average market intakes with all types of brands considered Scenario C			
Component	Gender	*p*‐Value	Mean	Min	Max	*SD*	Mean	Min	Max	*SD*	Mean	Min	Max	*SD*	Mean	Min	Max	*SD*
Energy value (kcal/day)	Male *N* = 776	.9088	746	6	2,391	13	745	6	2,446	13	739	7	2,448	13	752	6	2,431	13
	Female *N* = 1,142	.9187	634	60	2,030	8	632	63	1,987	8	629	57	1,988	8	638	62	2,001	8
Fats (g/day)	Male *N* = 776	.6333	29.9	0.2	100.1	0.6	30.5	0.2	103.2	0.6	30.6	0.3	104.7	0.6	30.6	0.2	103.5	0.6
	Female *N* = 1,142	.7933	24.4	2.3	98.9	0.3	24.7	1.8	96.8	0.4	24.7	1.8	96.9	0.4	24.9	2.3	97.3	0.3
Carbohydrates (g/day)	Male *N* = 776	.6806	94.6	1.0	343.8	2.0	92.3	0.9	344.9	2.0	93.2	0.9	350.1	2.0	94.7	1.0	344.0	2.0
	Female *N* = 1,142	.7000	82.8	6.8	369.8	1.2	81.5	6.5	357.8	1.2	82.5	6.3	378.6	1.3	83.2	6.7	377.8	1.3
Proteins (g/day)	Male *N* = 776	.0001	24.0 a	0.1	64.0	0.4	24.4 a	0.2	64.2	0.4	22.2 b	0.1	61.0	0.4	23.8	0.1	62.8	0.4
	Female *N* = 1,142	<.0001	20.1 a	1.4	64.1	0.2	20.2 a	1.1	64.0	0.3	18.7 b	1.2	58.0	0.2	19.9	1.3	63.7	0.2

Min, Minimum; Max, maximum; DS, standard deviation.

Proteins appeared to be the only nutrient for which there were significant differences between average daily intakes both for adult populations shown in Table 5, but also for teenagers and children (data not shown). Indeed, average daily protein intake from first‐price products (scenario A3), related to the consumption of the 343 foodstuffs studied, was significantly lower than that for national (scenario A1) and retailer brands (scenario A2), for all the populations studied. For instance, men's average daily protein intake was 22.2 g/100 g for first‐price products, 24.0 g/100 g for national brands, and 24.4 g/100 g for retailer brand products.

These results correspond to a theoretical simulation of individuals consuming exclusively processed first‐price products in the scope of a partial diet. They nonetheless suggest a tendency to lower protein intake through the consumption of first‐price products. This can be related to the significant differences and tendencies noted previously for proteins in the study of nutrition values according to the type of brand.

In the case of high‐loyalty scenarios (scenario [b]), no significant difference between average daily intakes was noted, whatever the nutrient considered (data not shown).

## 4 DISCUSSION

The aim of this study was to explore, on the large scale of almost complete coverage of the French processed foods market, nutrition differences between types of brand, and whether the differences that might be observed at product level were magnified or diminished at food intake level by crossing nutrition contents with consumption data. To our knowledge, this is the first study comparing nutrition quality between types of brands for such a large sample of processed foodstuffs (the estimated market coverages per type of brand all being around 70%). We decided to compare nutrition values nutrient by nutrient, rather than using some nutrition quality scoring method, in order to study each nutrient, and then nutrient intakes, separately and to be able to determine small and isolated differences. This means that our study did not take into account any concepts of recipe or source of ingredients.

This work shows that, when considering the nutrition values labeled on processed foods on the French market, only isolated and nonsystematic differences could be determined between types of brands. The absence of systematic differences in the nutrition contents of processed foods from various types of brands is an encouraging result when considering social inequalities and nutrition. It strongly validates the results obtained by previous and more specific studies that also compared nutrition values between types of brands. Such studies were carried out on restricted ranges of food groups and smaller numbers of products. Indeed, the French consumer review, “60 million consumers,” devoted an issue to this subject in 2007 (Guibert, 2007). Their investigation consisted of two comparative trials based on raviolis (22 products compared) and chocolate‐filled biscuits (22 products) from national brands, retailer brands, hard discount, and first‐price brands. Concerning raviolis, it was emphasized that national and retailer brand products generally contained more meat than first‐price ones: However, this could not be linked to a lower amount of proteins as they were also contained in the wheat used for the dough and the filling or in the egg whites in the dough.

In 2009, the CLCV similarly compared the nutrition information labeled on more than 300 discount, retailer brand, and national brand products (CLCV, 2009). Discount products did not appear to have a higher fat content or to be more rich in calories (compared to national and retailer brands): Sometimes, it was even the other way round. For the sample of products they studied, Cooper and Nelson (Cooper & Nelson, 2003) also showed that economy‐line foods (products cheap in price and plain in packaging) had a nutrient composition similar to and often better than the branded foods. Faulkner et al. (2014) compared the nutrition quality of supermarket own brand versus market brand foods in the UK market in 2010 and 2012. Their study was based on a limited range of products (32 food products from six food groups) representative of a UK consumer's shopping basket, and nutrient contents were compared using a nutrition quality scoring method based on the Food Standards Agency's Traffic Light System. No difference was found in overall nutrition quality between the market brand and the own brand food basket. A very similar Dutch study, collecting information from back‐of‐pack nutrition tables from 430 food products, led to the same findings (Waterlander et al., 2014).

It should be emphasized that the difficulty of studying labeled nutrition values is due in particular to the availability of such labeling. A study published in 2007 on first‐price foods as compared to branded ones (Joly et al., 2007) already stated the difficulty of such research due to often inadequate nutrition labeling, especially among first‐price products. In the present study, 94% of retailer brands, 90% of national brands versus 87% of hard discount, and 71% of entry‐level retailer brands labeled Big 4 component contents (energy, fats, proteins, and carbohydrates). Concerning Big 8 components (previous components plus sugars, fibers, sodium, saturated fatty acids), a wider gap was observed: 76% for retailer brands, 61% for national brands versus 41% for hard discount, and 28% for entry‐level retailer brands (Perrin et al., 2017). It must be pointed out that considered products were collected between 2008 and 2011, when EU regulation n°1169/2011, which makes detailed nutritional labeling mandatory, was not yet in force. For this study and the simulation of the potential impact on nutrient intake part, due to the lower nutrition labeling among first‐price products, it should also be noted that, among the 343 INCA 2 foodstuffs studied, numerous average nutrient content values per type of brand were calculated on less than three Oqali products.

The study eventually took into account 10,640 Oqali products, associated with 343 INCA 2 foodstuffs, out of the approximately 14,400 products labeling nutrition values. This difference is due both to the necessity of having the three types of brands represented for the INCA 2 foodstuffs studied and to the need for associated sales volumes in order to calculate weighted averages. The scope of the study has thus been reduced to cover only those references for which all of the necessary data are available. This limits the links one may draw between the two parts of this study. One should also note that, due to the fact that the INCA 2 foodstuffs studied represent only a part of the diet, this led, for instance, to higher energy intakes for young girls (693 kcal/day for all types of brands considered) than for women (638 kcal/day for all types of brands considered), due to the specific foodstuffs and food groups taken into account in this part of the study.

Finally, the tendency to lower protein intake due to the consumption of first‐price products that has been highlighted in this study should be qualified, as protein intake of the French population is currently above recommendations (Afssa, 2007): In this respect, such first‐price foodstuffs consumption does not imply any risk of deficiency or insufficient intake for French consumers.

## 5 CONCLUSIONS

Among the 16,081 processed products considered, collected from 24 food sectors between 2008 and 2011, and considering the nutrient contents labeled, only isolated and nonsystematic differences in the nutrient contents between types of brands were underlined. No cross‐sectional tendency was found among the 24 food sectors studied in this comparative study between types of brands. When crossing these labeled nutrition values with consumption data from the French INCA 2 study (Afssa, 2006) and in the case of a theoretical maximalist simulation of individuals consuming exclusively processed foodstuffs from one specific type of brand, protein intake from first‐price products appeared to be significantly lower than the ones from national brand or retailer brand products. No difference in nutrition intake could be observed in a less extreme scenario, where foodstuffs from one specific type of brand were just consumed predominantly and not exclusively. These results nonetheless suggest a tendency to lower protein intake due to the consumption of first‐price products. The absence of systematic differences in the nutrition contents of processed foods from various types of brands is an encouraging result when considering social inequalities and nutrition.

## CONFLICT OF INTEREST

The authors have no conflict of interests.

## AUTHOR CONTRIBUTION

CP and JGB were responsible for the conception and the design of this manuscript. The manuscript was written by CP and was revised by JGB, CM, and JLV.

## ENDNOTES

1 Representative household purchases data of the French population.

2 Nutrition labeling: display of the nutrition content of a food product, either by labeling the “Big 4” (energy, protein, carbohydrate, and fat) or the “Big 8” (energy, protein, carbohydrate, sugars, fat, saturated fat, fiber, and sodium/salt) per 100 g. Any additional nutrient amount can be labeled, for instance to support a nutrition claim.

3 Detailed nutrition labeling corresponds to at least a “Big 8” nutrition labeling, with possible additional nutrients labeled.
